# Functional outcome in psychotic and affective inpatients: role of cognitive function

**DOI:** 10.1192/j.eurpsy.2025.342

**Published:** 2025-08-26

**Authors:** E. I. Maihofer, G. Sachs, A. Erfurth

**Affiliations:** 1 1st Department of Psychiatry and Psychotherapeutic Medicine, Klinik Hietzing; 2 Medical University of Vienna, Vienna, Austria

## Abstract

**Introduction:**

Functional outcome is a central clinical concern in inpatient psychiatry. Neurocognition is known to be an important factor in achieving a good functional outcome.

**Objectives:**

We have previously investigated whether cognitive dysfunction improves over the course of inpatient treatment, where acutely admitted patients are offered a combination of pharmacological treatment and cognitive remediation (Maihofer et al. J Clin Med 2024, 13, 4843). We now investigate the extent to which the functional outcome of patients with psychotic and affective disorders is associated with cognitive function over time.

**Methods:**

Adult inpatients aged 18-66 years (female = 57.9%, male = 42.1%) were assessed with the Screen for Cognitive Impairment in Psychiatry (German version, SCIP-G: Sachs et al. Schiz Res Cogn 2021, 25, 100197; Sachs et al. Schiz Res Cogn 2022, 29, 100259). According to ICD-10 research criteria, 83 patients received an F2 diagnosis (schizophrenia, schizoaffective and delusional disorders), 61 patients met the criteria for bipolar disorder or mania (F30/F31) and 90 for depression (F32/F33). All patients received state-of-the-art pharmacotherapy and cognitive remediation using the COGPACK® software package version 6.06. Functioning was assessed using the Global Assessment of Function (GAF).

**Results:**

SCIP scores at baseline correlate significantly with SCIP scores at time point two (r=.74, p<.001). The SCIP at baseline is significantly correlated with patients’ functional level (r=.32, p=.01). The higher the baseline SCIP score, the higher the GAF score (r=.33, p=.01). The higher the GAF score at baseline, the higher the SCIP score at time 2 (r=.26, p=.039). The higher the SCIP score at time 2, the higher the GAF score at time 2 (r=.42, p<.001).

**Conclusions:**

During their stay in hospital, acutely admitted patients improved in function and neurocognition, regardless of their diagnostic classification. Functionality as measured by the GAF correlates significantly with cognitive ability as assessed by the SCIP-G.

**Disclosure of Interest:**

None Declared

